# The Relationship Between Reduced Glomerular Filtration Rate and Hearing Impairment: A Study Based on Western and Eastern Population

**DOI:** 10.3390/jcm15155859

**Published:** 2026-07-27

**Authors:** Mingdong Wang, Jing Cui, Yishan Zhang, Yongjun Quan, Yingwei Xie, Li Chen, Yongchen Jin, Xiaoxuan Bai, Yuexin Liu, Xiaobo Ma, Dongning Chen, Dan Liu, Hao Ping

**Affiliations:** 1 Department of Urology, Beijing Tongren Hospital, Capital Medical University, Beijing 100730, China; 2 Department of Physical Examination, Beijing Tongren Hospital, Capital Medical University, Beijing 100730, China; 3 Institute of Child and Adolescent Health, School of Public Health, Peking University, Beijing 100871, China; 4 National Health Commission Key Laboratory of Reproductive Health, Beijing 100191, China; 5 Department of Otolaryngology, Head and Neck Surgery, Beijing Tongren Hospital, Capital Medical University, Beijing 100730, China; 6 Beijing Advanced Innovation Center for Big Data-Based Precision Medicine, Beihang University and Capital Medical University, Beijing Tongren Hospital, Beijing 100730, China

**Keywords:** renal function, reduced glomerular filtration rate, hearing impairment, NHANES database, real-world evidence

## Abstract

**Objectives**: To explore potential strategies for the early detection of renal function impairment, we investigated the association between renal function impairment and hearing loss, as early-stage renal impairment often presents with few clinical manifestations, making it challenging to detect. **Methods**: We included data including western and eastern population for 3154 participants from the NHANES database in 2009–12 and 2015–18, and for 5958 participants in the Beijing Tongren hospital between January 2019 and January 2023, with a median follow-up of 30.0 months. Then, we comprehensively evaluated the relationship between renal function and hearing impairment, using linear regression, Logistics regression, Cox regression, and Mann–Whitney U test. **Results:** People with reduced eGFR were more likely to show speech-frequency hearing loss than those with normal eGFR (HR 1.54; 95% CI 1.20–1.97; *p* < 0.001). People with speech-frequency hearing loss were more likely to have reduced eGFR (HR 1.49; 1.18–1.89; *p* < 0.001). Hearing acuity in women with reduced eGFR may improve with the amelioration of renal function (OR 1.10; 1.01–1.20; *p* = 0.037). **Conclusions:** Renal function examinations may be considered in people with self-perceived or tested hearing loss to detect potential early renal injury. People with reduced eGFR should also place attention on hearing protection, given the observed association. Whether a causal relationship exists between hearing function and eGFR remains to be elucidated through further in-depth studies.

## 1. Introduction

Chronic kidney disease (CKD) ranks as the 16th leading cause of reduced lifespan worldwide, impacting about 8–16% of the global population. CKD brings a huge burden of medical costs to the healthcare system, and it is particularly prevalent among lower and middle-income groups [1,2].

CKD has been considered to have a connection with hearing impairment since Prof. A. Cecil Alport’s first description in 1927 of a case linking hereditary kidney disease with hearing loss [3]. Over the years, reports have also demonstrated associations between various other types of kidney diseases and hearing loss [4,5,6]. Several studies from the past decade also support this viewpoint. For instance, a cross-sectional study involving a database of 5266 individuals from South Korea found that patients with lower estimated glomerular filtration rate (eGFR) had a higher likelihood of experiencing hearing impairment (average bilateral hearing threshold > 40 dB) than did those with normal eGFR levels [7]. Similar conclusions were validated in China’s population-based CHARLS database, in which an independent association between declining kidney function and hearing loss was reported [8]. Furthermore, some small-scale studies have noted an increased prevalence of high-frequency hearing loss in patients with chronic kidney disease and in those undergoing dialysis for end-stage renal disease [9,10,11]. However, a large-scale longitudinal study in Japan involving 127,147 participants arrived at a contrasting conclusion, suggesting that low eGFR does not increase the risk of hearing loss at high or low frequencies [12]. In addition, a large retrospective longitudinal study showed that there was no statistically significant correlation between eGFR low baseline values and the risk of hearing loss [13].

Up to now, existing research has not entirely elucidated the connection between reduced eGFR and hearing loss. Previous studies investigating this association did not fully assess the situation across all ethnicities, and there were some ambiguities in the definition of hearing impairment in these studies, which may have led to biased conclusions. Hence, it is essential to develop a well-defined, high-quality, representative large sample study of a multi-ethnic population.

Considering the irreversible progression of CKD [14] and the adverse impact of hearing loss on patients’ quality of life [15], the goal of our research was to assess the relationship between hearing loss and renal function in a multi-ethnic population in order to provide new insights for early diagnosis and treatment of renal function impairment and hearing loss. 

## 2. Materials and Methods

### 2.1. Study Population

The data used in this study are publicly accessible on the NHANES website (https://wwwn.cdc.gov/nchs/nhanes/Default.aspx, accessed on 1 October 2023). To ensure an adequate sample size and wider age coverage, we collected data from participants aged 20 years or older who had complete hearing information and serum creatinine from the NHANES survey between 2009 and 2012 and between 2015 and 2018. We excluded those with incomplete demographic information, lifestyle information, or chronic-disease data.

The Beijing Tongren Hospital Cohort study was a retrospective cohort study of Chinese men and women aged 18 years or older who had undergone at least one comprehensive health examination annually at the Beijing Tongren Hospital [16,17,18]. This Hospital has been a nationally top medical institute especially in terms of hearing examination. We retrospectively collected data for participants included in this cohort study between 1 January 2019, and 1 January 2023. Then, we excluded individuals with incomplete demographic information, incomplete hearing information, and incomplete serum creatinine. Data acquisition and specific variables are described in the Supplementary Materials (Tables S7–S10). 

**Figure 1:**
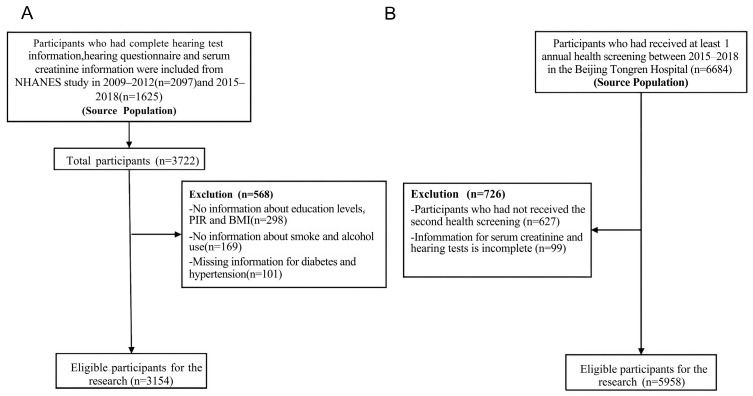
Flow diagram of participants’ inclusion. Study participants were derived from the NHANES database (**A**) and Tongren cohort (**B**). BMI = body mass index. PIR = family income-to-poverty ratio. eGFR = estimated glomerular filtration rate.

Of 3722 participants in the NHANES database, 3154 had complete data and were included from the cross-sectional study (Figure 1A). Among 6684 participants from the cohort study, 5958 participants were included in this study (Figure 1B).

### 2.2. Assessment of Renal Function with eGFR

The eGFR is the most commonly used indicator to assess renal function. In this study, eGFR was calculated with the Modification of Diet in Renal Disease study equation [19]: eGFR (mL/min/1.73 m^2^) = 175 × (serum creatinine [mg/dL]) − 1.154 × (age, years) − 0.203 × (0.742 if female) × (1.212 if African American).

Normal renal function was defined as an eGFR > 90 mL/min/1.73 m^2^. Patients with an eGFR between 60 and 90 mL/min/1.73 m^2^ were considered to have mildly declined renal function, while an eGFR persistently < 60 mL/min/1.73 m^2^ was defined as CKD [14].

### 2.3. Definition of Hearing Impairment

All participants received pure tone air conduction audiometry—a measurement of hearing sensitivity by presenting pure tone signals to the ear through earphones and by varying the intensity of the signals until a participant’s hearing threshold at that frequency is determined. Based on the average hearing threshold measured for bilateral ears at different frequencies, a hearing threshold exceeding 25 dB was defined as hearing impairment at that specific frequency [20]. 

Low-frequency hearing loss was defined as hearing impairment at 0.5, 1, or 2 kHz, while high-frequency hearing loss was defined as hearing impairment at 3, 4, or 6 kHz frequencies. Speech frequency is the most frequently heard hearing frequency in daily life, and speech-frequency hearing loss was defined as hearing impairment at 0.5, 1, 2, or 4 kHz frequency [21].

### 2.4. Covariates

In addition to eGFR and hearing data, our study incorporated other covariates. These covariates included gender, age, race, body mass index (BMI), family income-to-poverty ratio (PIR, comparing its total income to the official poverty threshold, adjusted for household size and composition), and educational level. To perform propensity score matching (PSM), we divided age into two groups (<60 years and ≥60 years), PIR into three groups (<1.3, 1.3–3.5, and >3.5), and BMI into three groups (<25 kg/m^2^, 25–30 kg/m^2^, and ≥30 kg/m^2^).

### 2.5. Statistical Analysis

Continuous variable data from the baseline characteristics table were expressed as mean (SD) based on the data distribution. We used the Mann–Whitney U test or *t*-test to compare numerical variables between the two groups. Categorical variable data were presented as frequency and percentage, and, after determining the data distribution, we compared categorical variables between groups using either the Chi-square test or Fisher’s exact test. 

We used generalized additive models and simple linear regression methods to assess the relationship between the risk of renal impairment and hearing loss. We applied univariate and multivariate logistic regression analyses to explore variables significantly associated with hearing impairment and to investigate whether CKD was an independent factor for hearing loss. 

We conducted a 1:1 PSM by using nearest-neighbor matching with a caliper width of 0.02, based on whether there was a decrease in eGFR (<90 mL/min/1.73 m^2^). For the matched groups, we used a Mann–Whitney U test to compare the differences in various frequency hearing functions between the groups, as well as the differences in self-perceived hearing impairment among different renal function groups. 

Univariate and multivariate Cox regression was used to explore the risk factors of the progression of hearing impairment in people with normal hearing in the first physical examination, and the risk factors for deterioration of renal function in people with normal renal function in the first physical examination. Then, logistic regression was used to investigate the protection factor of the improvement of hearing in participants with hearing impairment and simultaneously declined eGFR. A two-sided *p*-value lower than 0.05 was considered significant.

The plotting of graphs and data processing were performed using R software (version 4.0) and GraphPad Prism (version 8.0).

## 3. Results

### 3.1. Baseline Characteristics

The baseline characteristics of the candidates are presented in Tables S1 and S2. In the NHANES cohort, the mean values of eGFR in people with normal hearing were all above 90 mL/min/1.73 m^2^. Among individuals with hearing impairment, the mean eGFR values were all below 90 mL/min/1.73 m^2^. In the Tongren cohort, the mean eGFR was lower in people with speech-frequency hearing loss (94.8 mL/min/1.73 m^2^) than in people without hearing impairment (98.7 mL/min/1.73 m^2^).

Without adjusting for other variables, males seemed to be more likely to experience speech-frequency and high-frequency hearing impairment than females in the NANHES and Tongren cohorts (*p* < 0.05).

The level of education seemed to differ between patients with hearing impairment and those without (*p* < 0.05), with individuals receiving higher education exhibiting better hearing compared with those with lower educational attainment. 

Participants with hearing impairment appeared to be more susceptible to hypertension and diabetes than those without hearing impairment in the NHANES (*p* < 0.0001). In the Tongren cohort study, people with speech-frequency hearing loss had higher BMI (mean BMI, 25.6 kg/m^2^ [SD 3.53]) than those without hearing impairment (mean BMI, 24.4 kg/m^2^ [SD 4.09]; *p* < 0.001).

### 3.2. Association Between eGFR and Hearing Impairment

Generalized linear models and simple linear regression analyses showed that eGFR was inversely associated with hearing thresholds across low- and high-frequency ranges. This negative correlation became more obvious as the hearing frequency of the test increased (Figure S1). 

Univariate logistic regression and multivariate logistic regression analyses indicated that having eGFR levels lower than 60 mL/min/1.73 m^2^ was an independent risk factor for high-frequency hearing impairment (OR, 1.63; 95% CI, 1.09–2.44; *p* = 0.017) (Table S4) and for speech-frequency hearing impairment (OR, 1.91; 1.34–2.72; *p* < 0.001) (Table S5), but not for low-frequency hearing loss (OR, 1.31; 0.94–1.82; *p* = 0.110) (Table S3). 

Ageing was an independent risk factor for any hearing loss (Tables S3–S5). Furthermore, being Black and having a college education or higher were independent protection factors for any kind of hearing loss (Tables S3–S5). For speech-frequency hearing loss and high-frequency hearing loss, males were more likely to develop hearing loss (Tables S4 and S5). For high-frequency hearing loss, diabetes and noise exposure were independent risk factors for hearing impairment (Table S4). 

The baseline characteristics before and after matching are presented in Table S6. Before matching, there were differences in various baseline characteristics (*p* < 0.05, SMD > 0.1). However, after a 1:1 match, the variables achieved good balance (*p* > 0.5, SMD < 0.1). The process and results of matching can be found in Figures S2–S4.

We compared the hearing thresholds between individuals with decreased eGFR (<90 mL/min/1.73 m^2^) and those with normal eGFR in each frequency group after matching and found that individuals with decreased eGFR in 1 to 6 kHz exhibited poorer hearing levels (*p* < 0.05) (Figure S5). 

**Figure 2:**
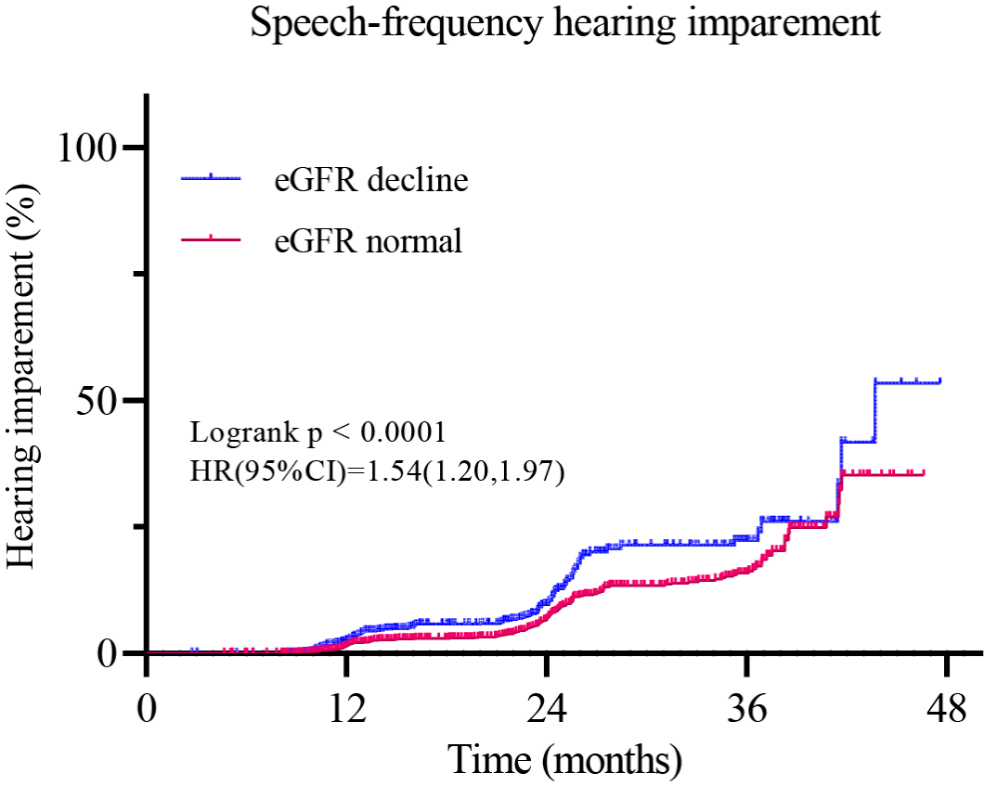
Comparison of the hearing status and the time of hearing loss between the eGFR decline group and the normal eGFR group. In the Tongren cohort study, participants without hearing loss at the beginning were divided into two groups according to whether there was a decrease in eGFR (<90 mL/min/1.73 m^2^; eGFR decline group) or not (normal eGFR group). eGFR = estimated glomerular filtration rate. HR = hazard ratio.

People without hearing loss from the Tongren cohort study were divided into two groups: the eGFR decline group (eGFR < 90 mL/min/1.73 m^2^) and the normal eGFR group (eGFR ≥ 90 mL/min/1.73 m^2^). Then, we collected whether and when they subsequently had hearing loss from the Tongren cohort data and drew a Kaplan–Meier curve. We found that people with low eGFR were more likely to develop speech-frequency hearing impairment (log-rank *p* < 0.0001; hazard ratio [HR] = 1.54; 95% CI, 1.20–1.97) (Figure 2). Furthermore, univariate and multivariate COX analysis showed that eGFR < 60 mL/min/1.73 m^2^ was an independent risk factor for hearing loss development (*p* < 0.001; hazard ratio [HR] = 4.07; 95% CI, 1.79–9.24) (Table S11).

### 3.3. Association of eGFR Decline and Self-Perceived Hearing Loss

**Figure 3:**
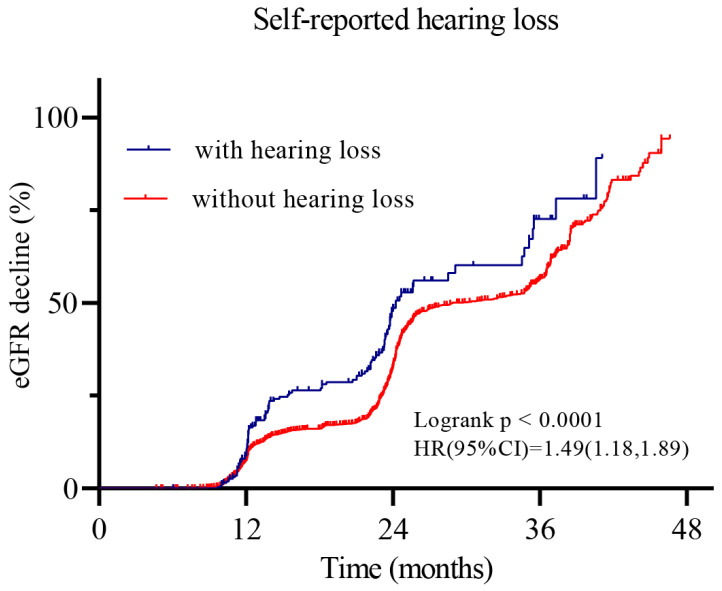
Comparison of time and occurrence of eGFR decline between the participants with perceived hearing loss and those without. In the Tongren cohort study, participants without eGFR decline at the beginning were divided into two groups according to whether they had or did not have perceived hearing loss. eGFR, estimated glomerular filtration rate; HR, hazard ratio.

We explored the relationship between self-perceived hearing impairment and eGFR levels and found a significant association between mild eGFR decline (eGFR in 60–90 mL/min/1.73 m^2^) and self-reported hearing loss (*p* < 0.001) (Figure S6). In the Tongren cohort study, we selected the participants with normal eGFR levels, divided them into two groups according to whether they had self-perceived hearing loss, calculated the time of their eGFR decline (eGFR < 90 mL/min/1.73 m^2^), and drew the Kaplan–Meier curve and conducted univariate and multivariate COX analysis. We found that people with self-perceived hearing loss were more likely to develop eGFR decline (log-rank *p* < 0.0001; HR = 1.49; 95% CI, 1.20–1.97) (Figure 3). We also concluded that self-perceived hearing loss was an independent risk factor for eGFR decline (*p* = 0.009; hazard ratio [HR] = 1.31; 95% CI, 1.07–1.61) (Table S12) and CKD (*p* = 0.002; hazard ratio [HR] = 1.49; 95% CI, 1.15–1.91) (Table S13).

### 3.4. Factors Related to Hearing Improvement

**Figure 4:**
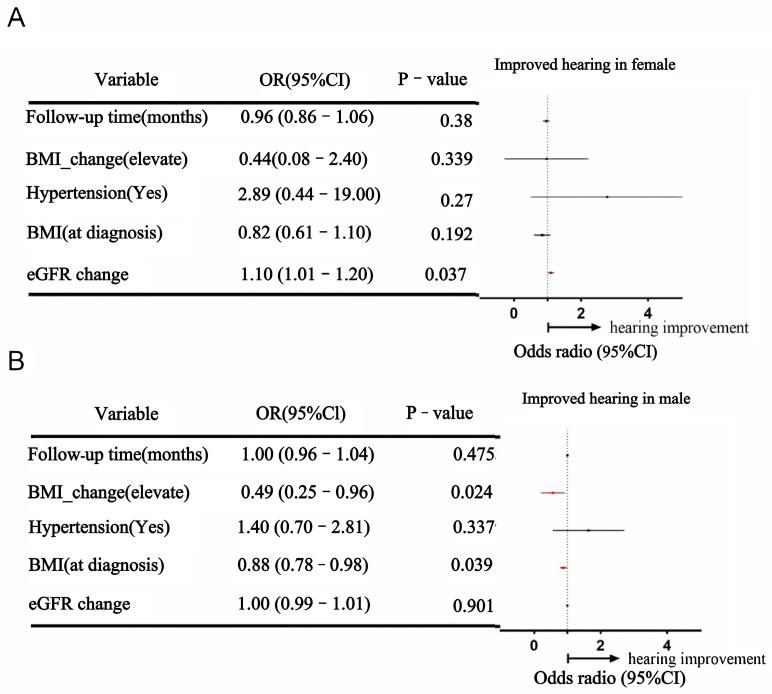
Forest plot of multivariate logistic regression of the protective factors of hearing improvement in participants with declined eGFR and hearing impairment. (**A**) In Chinese females, elevated eGFR is positively associated with improved hearing function (OR = 1.10, *p* = 0.037). (**B**) Low-weight Chinese males (OR = 0.88, *p* = 0.039) and losing weight (OR = 0.49, *p* = 0.024) are positively associated with improved hearing function.

Univariate and multivariate logistic regression showed that elevated eGFR in female with hearing loss is positively associated with improved hearing function (*p* = 0.037; odds ratio [OR]= 1.10; 95% CI, 1.01–1.20) (Table S14). However, this phenomenon was not consistent among males. In addition, low BMI of men negatively associated with improved hearing function (*p* = 0.024; odds ratio [OR] = 0.49; 95% CI, 0.25–0.96), and weight loss of men also positively associated with improved hearing function (*p* = 0.039; odds ratio [OR]= 0.88; 95% CI, 0.78–0.98) (Figure 4).

## 4. Discussion

To increase representativeness in our study, we combined large cross-sectional data from the NHANES database from the United States with retrospective cohort data collected from a Chinese hospital to explore the association between renal function and hearing impairment.

We used the same eGFR calculation method and definition of hearing loss in both cohorts to ensure the consistency of the research conclusions. Our research found that an eGFR of less than 60 mL/min/1.73 m^2^ is an independent risk factor for high-frequency and speech-frequency hearing impairments. In addition to ageing (a well-known factor affecting hearing) [22], sociodemographic factors such as college or higher education were an independent protective factor, showing a protective effect on hearing impairment at all frequencies tested [14]. Furthermore, consistent with previous study conclusions, being Black was an independent protective factor for hearing compared to other races [23], suggesting potential racial effects. Regarding gender, males showed a higher susceptibility to hearing impairment in speech-frequency and high-frequency hearing impairments than females, aligning with earlier research conclusions [23].

The retrospective study from the Blue Mountains Hearing Study in Australia in 2010 indicated that moderate CKD was an independent risk factor for hearing impairment, with over half of the participants with CKD having some degree of hearing loss [24]. In another prospective study assessing cochlear function in patients (aged 18–45 years) across different stages of CKD, 46% of patients with advanced CKD showed early cochlear dysfunction and subclinical hearing impairment [25]. This conclusion is consistent with our findings. However, in our study, we found that CKD was not an independent risk factor for low-frequency hearing impairment. This might be due to the progressive nature of hearing loss, which starts in high frequencies and ends in low frequencies; by the time patients have developed low-frequency hearing impairment, the impact of renal impairment on hearing may be masked by organic lesions in the cochlea or nervous system. 

We found that eGFR was negatively correlated with hearing thresholds, most notably at a hearing test frequency of 6 kHz (Figure 2). We further proved that people with reduced eGFR are more likely to develop hearing loss (Figure 3). As the test frequency for hearing decreases, the relative risk is weakened. Therefore, we speculate that decreased eGFR initially causes high-frequency hearing impairment, which then progresses to affect low-frequency hearing. This conclusion aligns with the known sequence of frequency involvement in hearing impairment [26]. 

In anatomy, there are significant similarities between the cochlea and the kidneys, such as similar physiological mechanisms between the stria vascularis in the cochlea and the glomerulus in the kidney [6]. Interestingly, many factors contributing to renal impairment are also common causes of hearing loss, such as hypertension, diabetes, and ageing [27,28], and most nephrotoxic drugs present with ototoxicity, including commonly used medications such as furosemide and aminoglycosides [29,30].

The pathophysiological mechanisms behind the hearing impairment caused by renal dysfunction are not yet fully understood. Current mainstream views attribute it to uremic neuropathy [31] and disturbances in body fluid and electrolytes [6,32,33]. Previous studies have observed alterations in both the peripheral and central nervous systems of patients with CKD, suggesting the existence of “uremic neuropathy” [34], and in the CKD patient population, there is a high incidence of neuronal conduction dysfunction. Several studies on auditory brainstem response in patients with CKD have demonstrated impairments in auditory nerves and pathways [6,32,35]. However, this theory does not fully explain high-frequency hearing loss resulting from early-stage renal impairment. 

Another theory holds that the hearing loss caused by renal impairment is attributed to the disorder of cochlear endolymph and the imbalance of electrolytes. Given the expression of many ion channels and transporters involved in K+ circulation, along with dynamic balances of K^+^, Na^+^, Ca^2+^, and pH in the inner ear and kidneys [36], researchers studying uremic rat models found reduced Na^+^–K^+^ ATPase activity in the experimental group [6]. Additionally, Yassin et al. [33] found a direct correlation between the degree of hearing loss and hyponatremia, unrelated to blood urea levels, and significant improvement in cochlear pathology was achieved by improving renal failure and restoring serum sodium levels. However, this theory does not fully elucidate the pathophysiological mechanisms; Bazzi et al. [9] assessed hearing loss in patients undergoing hemodialysis and concluded a high prevalence of hearing loss in these patients, independent of the duration of dialysis treatment itself.

Hearing impairment can be divided into conductive hearing loss and sensory hearing loss or sensorineural hearing loss. Previous studies on the relationship between renal damage and hearing loss mainly focused on sensorineural hearing loss. However, the association between renal damage and conductive hearing loss is not clear. Some studies have shown that abnormal bone metabolism (such as osteoporosis) can affect hearing by changing the bone mineral density of the cochlea and auditory ossicles [37,38]. Considering that renal function may be closely related to bone metabolism [39,40], we speculate that renal function damage can lead to conductive hearing loss by changing normal bone metabolism [41]. Therefore, whether the early improvement of renal function can help to delay or treat hearing loss by improving the health status of the cochlea and ossicles is still a question worth exploring. However, the NHANES database did not have enough data for the study population in terms of bone metabolism-related laboratory tests and bone mineral density, and therefore, we did not conduct a mediating effect analysis to explore the relationship between these factors and hearing loss.

The clinical presentations of CKD were fatigue, poor appetite, nausea, vomiting, metallic taste, unintentional weight loss, pruritus, changes in mental status, dyspnea, or peripheral edema, most of which occurred in the CKD late stages. However, people with early renal function impairment (eGFR of 60–90 mL/min/1.73 m^2^) had no typical clinical manifestations. Across all age groups, GFR levels lower than 60 mL/min/1.73 m^2^ have been associated with adverse outcomes, especially cardiovascular events and all-cause mortality [42]. However, the latest research shows that for people older than 65 years (reference GFR of 75–89 mL/min/1.73 m^2^), the risk of mortality is minimal until eGFR falls below 45 mL/min/1.73 m^2^. In the youngest age group (18–54 years old; reference eGFR of 105 mL/min/1.73 m^2^), the risk of mortality begins to increase when eGFR is below 75 mL/min/1.73 m^2^. Compared with the decline in renal function caused by age in older people, the mortality rate caused by a mild decrease in renal function in young people is higher than that in older people [42]. Therefore, it is very important to identify early renal dysfunction. Our study found that self-perceived hearing loss can be the clinical manifestation of some patients with mild renal impairment (Figure S6). People with self-perceived hearing loss were prone to get eGFR decline (<90 mL/min/1.73 m^2^) (Figure 4). Therefore, we speculate that for some people, hearing loss may indicate early renal function damage. For such patients, early identification of renal function damage could improve prognosis. Attention should be paid to the hearing status of patients with CKD from early stage in order to implement appropriate intervention early to reduce the burden of the disease. 

We examined the relationship between eGFR changes and hearing improvement. Elevated eGFR was positively associated with hearing improvement in Chinese women, but this association was not prominent in men. Notably, low BMI and weight loss were both positively correlated with hearing improvement in male patients, offering new directions for hearing loss intervention. 

Our study combines large cross-sectional data and retrospective cohort data from two different and representative countries with population of different ethnicities, which improves its representativeness. Moreover, we used the same eGFR calculation and definition of hearing loss in both cohorts to ensure consistency. 

However, our study has some limitations. Renal function was estimated using the MDRD equation rather than the CKD-EPI equation. CKD-EPI generally provides better GFR estimation than MDRD, particularly at higher eGFR levels, whereas MDRD may underestimate GFR and lead to potential misclassification of renal function. Future studies using CKD-EPI-based eGFR are warranted to validate our findings. A frequency-specific cutoff of >25 dB HL cannot fully capture actual hearing loss severity, which should ideally be graded by pure-tone average (mild to profound), and future work should explore differential links across hearing loss severity levels. Age was categorized only as <60 and ≥60 years, which may have introduced within-group heterogeneity and residual age-related confounding. Future studies with larger samples and more refined age stratification are needed to further validate our findings. Although diabetes mellitus, hypertension, and noise exposure were included in the univariate and multivariate analyses, data on ototoxic drug use and the severity or control status of systemic diseases were unavailable. Therefore, residual confounding cannot be fully excluded, and future studies should further address these factors.

## 5. Conclusions

For the first time, we found that one of the clinical manifestations of some patients with early renal dysfunction was self-perceived hearing loss. Early renal function examination in patients with self-perceived hearing loss should be performed to prevent possible renal damage. In Chinese women with reduced eGFR, improvement in hearing is correlated with improvement in renal function, indicating an association between these two conditions. However, whether a causal relationship exists between hearing and eGFR remains to be elucidated through further in-depth studies. 

## Data Availability

The cross-sectional data were obtained from the NHANES database. Raw data of Tongren Cohort and the detailed protocol are available upon reasonable request to the corresponding author of Hao Ping (pinghaotrh@ccmu.edu.cn) or Dan Liu (drliudan@163.com).

## Supplementary Materials

The following supporting information can be downloaded at https://www.mdpi.com/article/10.3390/jcm15155859/s1, Table S1. Demographic and socio-behavioral characteristics of the study population from NHANES database; Table S2. Demographic and socio-behavioral characteristics of the study population from Tongren cohort data; Table S3. Univariate logistic regression and multivariate logistic regression of the association between variables and low-frequency hearing loss; Table S4. Univariate logistic regression and multivariate logistic regression of the association between variables and high-frequency hearing loss; Table S5. Univariate logistic regression and multivariate logistic regression of the association between variables and speech-frequency hearing loss; Table S6. Baseline characteristics of the participants before and after propensity score matching; Table S7. Hearing test of NHANES database; Table S8. Hearing related questionnaires of NHANES database; Table S9. Other variables of NHANES database; Table S10. Hearing questionnaire and hearing test of Tongren cohort; Table S11. Univariate Cox regression and multivariate Cox regression of the association between variables and eGFR decline in Tongren cohort; Table S12. Univariate Cox regression and multivariate Cox regression of the association between variables and eGFR decline in Tongren cohort; Table S13. Univariate Cox regression and multivariate Cox regression of the association between variables and CKD in Tongren cohort; Table S14. Association between hearing improvement and eGFR change among Chinese adults in Tongren cohort; Figure S1. Association between eGFR and hearing threshold in different frequency hearing tests based on NHANES database; Figure S2. Absolute standardized mean difference of the process of propensity score matching in the NHANES population; Figure S3. Distribution of propensity scores of unmatched and matched units; Figure S4. Proportion of the propensity score of unmatched and matched group; Figure S5. Comparison of binaural average hearing loss threshold at different frequency hearing test thresholds after propensity score matching based on whether eGFR was decreased; Figure S6. Comparison of different eGFR statuses in NHANES participants with and without self-perceived hearing loss.

## Abbreviations

The following abbreviations are used in this manuscript:

CKD	chronic kidney disease
eGFR	estimated glomerular filtration rate
BMI	body mass index
PIR	income-to-poverty ratio
