# Supplementary material for: The Relationship Between Reduced Glomerular Filtration Rate and Hearing Impairment: A Study Based on Western and Eastern Population
Source: J Clin Med. 2026 Jul 27;15(15):5859. doi: 10.3390/jcm15155859 (PMC13467505; doi:10.3390/jcm15155859)
Supplement: Supplementary file 1 [file jcm-15-05859-s001.zip › jcm-4352708-supplementary.pdf]

The Relationship Between Reduced Glomerular Filtration Rate and Hearing Impairment: A Study Based on Western and Eastern Population

Table S1. Demographic and socio-behavioral characteristics of the study population from NHANES database.
Table S2. Demographic and socio-behavioral characteristics of the study population from Tongren cohort data.
Table S3. Univariate logistic regression and multivariate logistic regression of the association between variables and Low-frequency hearing loss.
Table S4. Univariate logistic regression and multivariate logistic regression of the association between variables and High-frequency hearing loss.
Table S5. Univariate logistic regression and multivariate logistic regression of the association between variables and Speech-frequency hearing loss.
Table S6. Baseline characteristics of the participants before and after propensity score matching.
Table S7. Hearing test of NHANES database.
Table S8. Hearing related questionnaires of NHANES database.
Table S9. Other variables of NHANES database.
Table S10. Hearing questionnaire and hearing test of Tongren cohort.
Table S11. Univariate Cox regression and multivariate Cox regression of the association between variables and eGFR decline in Tongren cohort.
Table S12. Univariate Cox regression and multivariate Cox regression of the association between variables and eGFR decline in Tongren cohort.
Table S13. Univariate Cox regression and multivariate Cox regression of the association between variables and CKD in Tongren cohort.
Table S14. Association between hearing improvement and eGFR change among Chinese adults in Tongren cohort.

Figure S1. Association between eGFR and hearing threshold in different frequency hearing tests based on NHANES database.
Figure S2. Absolute standardized mean difference of the process of propensity score matching in the NHANES population.
Figure S3. Distribution of propensity scores of unmatched and matched units.
Figure S4. Proportion of the propensity score of unmatched and matched group.
Figure S5. Comparison of binaural average hearing loss threshold at different frequency hearing test thresholds after propensity score matching based on whether eGFR was decreased
Figure S6. Comparison of different eGFR statuses in NHANES participants with and without self-perceived hearing loss.

Table S1. Demographic and socio-behavioral characteristics of the study population from NHANES database

Variable		LFHL			SFHL			HFHL		
		Without LFHL	LFHL		Without SFHL	SFHL		Without HFHL	HFHL	
		(N=2397)	(N=757)	P.value	(N=2000)	(N=1154)	P.value	(N=1528)	(N=1626)	P.value
Age (years)										
	Mean (SD)	48.7 (17.1)	71.7 (10.8)	<0.001	45.3 (16.0)	69.6 (11.4)	<0.001	41.4 (14.6)	66.2 (13.2)	<0.001
	Median [IQR]	48.0 [34.0–62.0]	75.0 [71.0–80.0]		44.0 [32.0–58.0]	73.0 [63.0–80.0]		39.0 [30.0–52.0]	70.0 [58.0–77.0]	
Sex										
	Female	796 (33.2%)	274 (36.2%)	0.142	721 (36.1%)	349 (30.2%)	0.00104	605 (39.6%)	465 (28.6%)	<0.001
	Male	1601 (66.8%)	483 (63.8%)		1279 (64.0%)	805 (69.8%)		923 (60.4%)	1161 (71.4%)	
Race										
	Non-Hispanic White	1061 (44.3%)	486 (64.2%)	<0.001	819 (41.0%)	728 (63.1%)	<0.001	595 (38.9%)	952 (58.5%)	<0.001
	Non-Hispanic Black	552 (23.0%)	101 (13.3%)		507 (25.4%)	146 (12.7%)		412 (27.0%)	241 (14.8%)	
	Mexican American	338 (14.1%)	80 (10.6%)		284 (14.2%)	134 (11.6%)		215 (14.1%)	203 (12.5%)	
	Other Hispanic	243 (10.1%)	42 (5.5%)		212 (10.6%)	73 (6.3%)		168 (11.0%)	117 (7.2%)	
	Other Race	203 (8.5%)	48 (6.3%)		178 (8.9%)	73 (6.3%)		138 (9.0%)	113 (6.9%)	
PIR										
	<1.3	817 (34.1%)	218 (28.8%)	0.00148	696 (34.8%)	339 (29.4%)	0.00333	584 (35.9%)	487 (30.0%)	0.0018
	1.3–3.5	996 (41.6%)	370 (48.9%)		827 (41.4%)	539 (46.7%)		629 (41.2%)	737 (45.3%)	
	⩾3.5	584 (24.4%)	169 (22.3%)		477 (23.9%)	276 (23.9%)		351 (23.0%)	402 (24.7%)	
Education										
	Less than high school	564 (23.5%)	265 (35.0%)	<0.001	440 (22.0%)	389 (33.7%)	<0.001	317 (20.7%)	512 (31.5%)	<0.001
	High-Grad/GED	649 (27.1%)	196 (25.9%)		545 (27.3%)	300 (26.0%)		418 (27.4%)	427 (26.3%)	
	Some College/AA	808 (33.7%)	183 (24.2%)		689 (34.5%)	302 (26.2%)		546 (35.7%)	455 (27.4%)	
	College/above	376 (15.7%)	113 (14.9%)		326 (16.3%)	163 (14.1%)		247 (16.2%)	242 (14.9%)	
BMI (kg/m^2)										
	<25	575 (24.0%)	195 (25.8%)	0.325	490 (24.5%)	280 (24.3%)	0.199	395 (25.9%)	275 (23.1%)	0.0446
	25–30	816 (34.0%)	267 (35.3%)		665 (33.3%)	418 (36.2%)		494 (32.3%)	589 (36.2%)	
	⩾30	1006 (42.0%)	295 (39.0%)		845 (42.3%)	456 (39.5%)		639 (41.8%)	662 (40.7%)	

Diabetes										
	No	2047 (85.4%)	560 (74.0%)	<0.001	1757 (87.9%)	850 (73.7%)	<0.001	1384(90.8%)	1220 (75.0%)	<0.001
	Yes	350 (14.6%)	197 (26.0%)		243 (12.2%)	304 (26.3%)		141 (9.2%)	406 (25.0%)	
Hypertension										
	No	1455 (60.7%)	261 (34.5%)	<0.001	1278 (63.9%)	438 (38.0%)	<0.001	1046 (68.5%)	670 (41.2%)	<0.001
	Yes	942 (39.3%)	496 (65.5%)		722 (36.1%)	716 (62.0%)		482 (31.5%)	956 (58.8%)	
Alcohol										
	No	535 (22.3%)	294 (38.8%)	<0.001	431 (21.6%)	398 (34.5%)	<0.001	329 (21.5%)	500 (30.8%)	<0.001
	Yes	1862 (77.7%)	463 (61.2%)		1569 (78.5%)	756 (65.5%)		1199 (78.5%)	1126 (69.2%)	
Smoke										
	No	1094 (45.6%)	326 (43.1%)	0.23	966 (48.3%)	454 (39.3%)	<0.001	761 (49.8%)	659 (40.5%)	<0.001
	Yes	1303 (54.4%)	431 (56.9%)		1034 (51.7%)	700 (60.7%)		767 (50.2%)	967 (59.5%)	
Gun exposure										
	No	1094 (45.6%)	383 (50.6%)	0.0193	950 (47.5%)	527 (45.7%)	0.339	746(48.8%)	731 (45.0%)	0.0325
	Yes	1303 (54.4%)	374 (49.4%)		1050 (52.5%)	627 (54.3%)		782 (51.2%)	895 (55.0%)	
Work noise exposure										
	No	956 (39.9%)	373 (49.3%)	<0.001	798 (39.9%)	531 (46.0%)	<0.001	607 (39.7%)	722 (44.4%)	0.00871
	Yes	1441 (60.1%)	384 (50.7%)		1202 (60.1%)	623 (54.0%)		921 (60.3%)	904 (55.6%)	
Life noise exposure										
	No	1857 (77.5%)	624 (82.4%)	0.00434	1559 (78.0%)	922 (79.9%)	0.215	1184 (77.5%)	1297 (79.8%)	0.129
	Yes	540 (22.5%)	133 (17.6%)		441 (22.1%)	232 (20.1%)		344 (22.5%)	329 (20.2%)	
eGFR (mL/min/1.73 m²)										
	>90	1139 (47.5%)	142 (18.8%)	<0.001	1047 (52.4%)	234 (20.3%)	<0.001	800 (57.6%)	401 (24.7%)	<0.001
	60–90	1075 (44.8%)	380 (50.2%)		850 (42.5%)	605 (52.4%)		589 (38.5%)	866 (53.3%)	
	<60	183 (7.6%)	235 (31.0%)		103 (5.2%)	315 (27.3%)		59 (3.9%)	359 (22.1%)	
eGFR_numeric (mL/min/1.73 m²)										
	Mean (SD)	90.9 (24.9)	71.4 (23.8)	<0.001	93.7 (24.5)	73.3 (23.3)	<0.001	96.5 (24.3)	76.6 (23.7)	<0.001

Median [IQR]	88.6 [75.0–105]	69.3 [55.6–84.4]	91.4 [78.0–107]	71.5 [58.2–86.4]	88.2 [80.6–109]	77.2 [61.8–89.8]

AA=Associate in Arts. BMI=body-mass index. PIR=family income-to-poverty ratio. eGFR=estimated glomerular filtration rate. GED=General Educational Development. LFHL=low-frequency hearing loss. SFHL=speech-frequency hearing loss. HFHL=high-frequency hearing loss.

Table S2. Demographic and socio-behavioral characteristics of the study population from Tongren cohort data

Variable		SFHL Without SFHL (N=4688)	SFHL (N=1270)	*P* value
Age (years)				
	Mean (SD)	38.3 (8.89)	48.5 (11.0)	<0.001
	Median [IQR]	37.0 [32.0–43.0]	49.0 [40.0–57.0]	
Sex				
	Female	1998 (42.6%)	193 (15.2%)	<0.001
	Male	2690 (57.4%)	1077 (84.8%)	
BMI (kg/m²)				
	Mean (SD)	24.4 (4.09)	25.6 (3.53)	<0.001
	Median [IQR]	24.0 [21.4–26.8]	25.5 [23.3–27.6]	
Follow-up time(months)				
	Mean (SD)	19.9 (8.30)	19.5 (8.13)	0.0689
	Median [IQR]	21.6 [4.83–46.6]	20.1 [12.1–24.2]	
eGFR (mL/min/1.73 m²)				
	>90	3300 (70.4%)	764 (60.2%)	<0.001
	60~90	1327 (28.3%)	482 (38.0%)	
	<60	61 (1.3%)	24 (1.9%)	
eGFR_numeric (mL/min/1.73 m²)				
	Mean (SD)	98.7 (19.3)	94.8 (18.9)	<0.001
	Median [IQR]	98.2 [88.0–110]	94.4 [84.1–105]	
Noise exposure				
	Yes	82 (1.7%)	59 (4.6%)	<0.001
	No	4606 (98.3%)	1211 (95.4%)	
Self-reported hearing loss				
	Yes	197 (4.2%)	234 (18.4%)	<0.001
	No	4491 (95.8%)	1036 (81.6%)	

BMI=body-mass index. PIR=family income-to-poverty ratio. eGFR=estimated glomerular filtration rate. SFHL=speech-frequency hearing loss.

Table S3. Univariate logistic regression and multivariate logistic regression of the association between variables and Low-frequency hearing loss

AA=Associate in Arts. BMI=body-mass index. PIR=family income-to-poverty ratio. eGFR=estimated glomerular filtration rate. GED=General Educational Development. OR=odds ratio. CI=confidence interval.

Variable		Low-frequency hearing impairment		univariable OR (CI95%, *P* value)	multivariable OR (CI95%, *P* value)
		No (N=2397)	Yes (N=757)		
Age (years)	Mean ± SD	48.7 ± 17.1	71.7 ± 10.8	1.12 (1.11-1.13, p<0.001)	1.11 (1.10-1.12, p<0.001)
PIR	<1.3	817 (34.1%)	218 (28.8%)		
	1.3–3.5	996 (41.6%)	370 (48.9%)	1.39 (1.15-1.69, p<0.001)	0.96 (0.75-1.23, p=0.730)
	≥3.5	584 (24.4%)	169 (22.3%)	1.08 (0.86-1.36, p=0.484)	0.77 (0.57-1.04, p=0.088)
BMI (kg/m²)	<25	575 (24%)	195 (25.8%)		
	25–30	816 (34%)	267 (35.3%)	0.96 (0.78-1.19, p=0.742)	
	≥30	1006 (42%)	295 (39%)	0.86 (0.70-1.06, p=0.171)	
Education	Less than high school	564 (23.5%)	265 (35%)		
	High-Grad/GED	649 (27.1%)	196 (25.9%)	0.64 (0.52-0.80, p<0.001)	0.79 (0.59-1.04, p=0.095)
	Some College/AA	808 (33.7%)	183 (24.2%)	0.48 (0.39-0.60, p<0.001)	0.60 (0.45-0.79, p<0.001)
	College/above	376 (15.7%)	113 (14.9%)	0.64 (0.50-0.83, p<0.001)	0.64 (0.45-0.90, p=0.0104)
Race	Mexican American	338 (14.1%)	80 (10.6%)		
	Non-Hispanic Black	552 (23%)	101 (13.3%)	0.77 (0.56-1.07, p=0.118)	0.64 (0.44-0.95, p=0.027)
	Non-Hispanic White	1061 (44.3%)	486 (64.2%)	1.94 (1.48-2.53, p<0.001)	1.19 (0.84-1.67, p=0.330)
	Other Hispanic	243 (10.1%)	42 (5.5%)	0.73 (0.49-1.10, p=0.131)	0.81 (0.50-1.30, p=0.377)
	Other Race	203 (8.5%)	48 (6.3%)	1.00 (0.67-1.49, p=0.996)	1.23 (0.75-2.01, p=0.417)
Sex	Female	796 (33.2%)	274 (36.2%)		
	Male	1601 (66.8%)	483 (63.8%)	0.88 (0.74-1.04, p=0.130)	
Diabetes	No	2047 (85.4%)	560 (74%)		
	Yes	350 (14.6%)	197 (26%)	2.06 (1.69-2.51, p<0.0001)	1.04 (0.81-1.32, p=0.781)
Smoke	No	1094 (45.6%)	326 (43.1%)		
	Yes	1303 (54.4%)	431 (56.9%)	1.11 (0.94-1.31, p=0.214)	
Hypertension	No	1455 (60.7%)	261 (34.5%)		
	Yes	942 (39.3%)	496 (65.5%)	2.94 (2.47-3.48, p<0.001)	1.10 (0.88-1.37, p=0.413)
Alcohol	No	535 (22.3%)	294 (38.8%)		
	Yes	1862 (77.7%)	463 (61.2%)	0.45 (0.38-0.54, p<0.001)	1.06 (0.84-1.33, p=0.635)
eGFR (ml/min/1.73m²)	>90	1139 (47.5%)	142 (18.8%)		
	60–90	1075 (44.8%)	380 (50.2%)	2.84 (2.30-3.50, p<0.001)	0.92 (0.71-1.20, p=0.559)
	<60	183 (7.6%)	235 (31%)	10.30 (7.94-13.36, p<0.001)	1.31 (0.94-1.82, p=0.110)
Noise exposure	No	487 (20.3%)	238 (31.4%)		
	Yes	1910 (79.7%)	519 (68.6%)	0.56 (0.46-0.67, p<0.001)	1.24 (0.98-1.56, p=0.079)

Table S4. Univariate logistic regression and multivariate logistic regression of the association between variables and High-frequency hearing loss

Variable		High-frequency hearing impairment		univariable OR (CI95%, *P* value)	multivariable OR (CI95%, *P* value)
		No (N=1528)	Yes (N=1626)		
Age (years)	Mean ± SD	41.4 ± 14.6	66.2 ± 13.2	1.11 (1.10-1.12, p<0.001)	1.13 (1.12-1.14, p<0.001)
PIR	<1.3	548 (35.9%)	487 (30%)		
	1.3–3.5	629 (41.2%)	737 (45.3%)	1.32 (1.12-1.55, p<0.001)	0.91 (0.72-1.16, p=0.449)
	≥3.5	351 (23%)	402 (24.7%)	1.29 (1.07-1.56, p=0.008)	0.80 (0.60-1.08, p=0.141)
BMI (kg/m²)	<25	395 (25.9%)	375 (23.1%)		
	25–30	494 (32.3%)	589 (36.2%)	1.26 (1.04-1.51, p=0.016)	1.13 (0.86-1.50, p=0.381)
	≥30	639 (41.8%)	662 (40.7%)	1.09 (0.91-1.30, p=0.337)	1.37 (1.04-1.82, p=0.027)
Education	Less than high school	317 (20.7%)	512 (31.5%)		
	High-Grad/GED	418 (27.4%)	427 (26.3%)	0.63 (0.52-0.77, p<0.001)	0.83 (0.62-1.10, p=0.192)
	Some College/AA	546 (35.7%)	445 (27.4%)	0.50 (0.42-0.61, p<0.001)	0.58 (0.44-0.78, p<0.001)
	College/above	247 (16.2%)	242 (14.9%)	0.61 (0.48-0.76, p<0.001)	0.49 (0.34-0.71, p=0.001)
Race	Mexican American	215 (14.1%)	203 (12.5%)		
	Non-Hispanic Black	412 (27%)	241 (14.8%)	0.62 (0.48-0.79, p<0.001)	0.37 (0.26-0.53, p<0.001)
	Non-Hispanic White	595 (38.9%)	952 (58.5%)	1.69 (1.36-2.11, p<0.001)	1.32 (0.94-1.83, p=0.105)
	Other Hispanic	168 (11%)	117 (7.2%)	0.74 (0.54-1.00, p=0.050)	0.68 (0.45-1.03, p=0.071)
	Other Race	138 (9%)	113 (6.9%)	0.87 (0.63-1.19, p=0.374)	1.04 (0.66-1.65, p=0.855)
Sex	Female	605 (39.6%)	465 (28.6%)		
	Male	923 (60.4%)	1161 (71.4%)	1.64 (1.41-1.90, p<0.001)	4.62 (3.56-5.99, p<0.001)
Diabetes	No	1387 (90.8%)	1220 (75%)		
	Yes	141 (9.2%)	406 (25%)	3.27 (2.66-4.02, p<0.001)	1.49 (1.12-1.99, p=0.006)
Smoke	No	761 (49.8%)	659 (40.5%)		
	Yes	767 (50.2%)	967 (59.5%)	1.46 (1.26-1.68, p<0.001)	0.93 (0.75-1.16, p=0.543)
Hypertension	No	1046 (68.5%)	670 (41.2%)		
	Yes	482 (31.5%)	956 (58.8%)	3.10 (2.68-3.58, p<0.001)	0.88 (0.70-1.11, p=0.283)
Alcohol	No	329 (21.5%)	500 (30.8%)		
	Yes	1199 (78.5%)	1126 (69.2%)	0.62 (0.53-0.73, p<0.001)	1.29 (0.99-1.69, p=0.061)
eGFR (ml/min/1.73m²)	>90	880 (57.6%)	401 (24.7%)		
	60–90	589 (38.5%)	866 (53.3%)	3.23 (2.76-3.78, p<0.001)	1.12 (0.89-1.40, p=0.336)
	<60	59 (3.9%)	359 (22.1%)	13.35 (9.90-18.02, p<0.001)	1.63 (1.09-2.44, p=0.017)
Noise exposure	No	321 (21%)	404 (24.8%)		
	Yes	1207 (79%)	1222 (75.2%)	0.80 (0.68-0.95, p=0.010)	1.35 (1.03-1.78, p=0.031)

AA=Associate in Arts. BMI=body-mass index. PIR=family income-to-poverty ratio. eGFR=estimated glomerular filtration rate. GED=General Educational Development. OR=odds ratio. CI=confidence interval.

Table S5. Univariate logistic regression and multivariate logistic regression of the association between variables and Speech-frequency hearing loss

AA=Associate in Arts. BMI=body-mass index. PIR=family income-to-poverty ratio. eGFR=estimated glomerular filtration rate. GED=General Educational Development. OR=odds ratio. CI=confidence interval

Variable		Speech-frequency hearing impairment		univariable OR (CI95%, *P* value)	multivariable OR (CI95%, *P* value)
		No (N=2000)	Yes (N=1154)		
Age (years)	Mean ± SD	45.3 ± 16.0	69.6 ± 11.4	1.12 (1.11-1.13, p<0.001)	1.12 (1.11-1.13, p<0.001)
PIR	<1.3	696 (34.8%)	339 (29.4%)		
	1.3–3.5	827 (41.4%)	539 (46.7%)	1.34 (1.13-1.58, p<0.001)	0.90 (0.71-1.15, p=0.394)
	≥3.5	477 (23.9%)	276 (23.9%)	1.19 (0.98-1.45, p=0.087)	0.82 (0.61-1.10, p=0.178)
BMI (kg/m²)	<25	490 (24.5%)	280 (24.3%)		
	25–30	665 (33.2%)	418 (36.2%)	1.10 (0.91-1.33, p=0.328)	
	≥30	845 (42.2%)	456 (39.5%)	0.94 (0.78-1.14, p=0.546)	
Education	Less than high school	440 (22%)	389 (33.7%)		
	High-Grad/GED	545 (27.2%)	300 (26%)	0.62 (0.51-0.76, p<0.001)	0.76 (0.57-1.01, p=0.058)
	Some College/AA	689 (34.5%)	302 (26.2%)	0.50 (0.41-0.60, p<0.001)	0.56 (0.42-0.74, p<0.001)
	College/above	326 (16.3%)	163 (14.1%)	0.57 (0.45-0.71, p<0.001)	0.40 (0.28-0.57, p<0.001)
Race	Mexican American	284 (14.2%)	134 (11.6%)		
	Non-Hispanic Black	507 (25.4%)	146 (12.7%)	0.61 (0.46-0.80, p<0.001)	0.40 (0.28-0.58, p<0.001)
	Non-Hispanic White	819 (41%)	728 (63.1%)	1.88 (1.50-2.37, p<0.001)	1.37 (0.99-1.90, p=0.061)
	Other Hispanic	212 (10.6%)	73 (6.3%)	0.73 (0.52-1.02, p=0.066)	0.77 (0.50-1.18, p=0.225)
	Other Race	178 (8.9%)	73 (6.3%)	0.87 (0.62-1.22, p=0.421)	1.05 (0.66-1.68, p=0.833)
Sex	Female	721 (36%)	349 (30.2%)		
	Male	1279 (64%)	805 (69.8%)	1.30 (1.11-1.52, p<0.001)	2.79 (2.15-3.60, p<0.001)
Diabetes	No	1757 (87.8%)	850 (73.7%)		
	Yes	243 (12.2%)	304 (26.3%)	2.59 (2.14-3.12, p<0.001)	1.28 (0.99-1.65, p=0.059)
Smoke	No	966 (48.3%)	454 (39.3%)		
	Yes	1034 (51.7%)	700 (60.7%)	1.44 (1.24-1.67, p<0.001)	1.12 (0.90-1.40, p=0.295)
Hypertension	No	1278 (63.9%)	438 (38%)		
	Yes	722 (36.1%)	716 (62%)	2.89 (2.49-3.36, p<0.001)	0.93 (0.75-1.16, p=0.531)
Alcohol	No	431 (21.6%)	398 (34.5%)		
	Yes	1569 (78.4%)	756 (65.5%)	0.52 (0.44-0.61, p<0.001)	1.07 (0.83-1.38, p=0.610)
eGFR (ml/min/1.73m²)	>90	1047 (52.4%)	234 (20.3%)		
	60–90	850 (42.5%)	605 (52.4%)	3.18 (2.67-3.80, p<0.001)	1.10 (0.86-1.40, p=0.439)
	<60	103 (5.2%)	315 (27.3%)	13.68 (10.51-17.81, p<0.001)	1.91 (1.34-2.72, p<0.001)
Noise exposure	No	416 (20.8%)	309 (26.8%)		
	Yes	1584 (79.2%)	845 (73.2%)	0.72 (0.61-0.85, p<0.001)	1.26 (0.96-1.65, p=0.092)

Table S6. Baseline characteristics of the participants before and after propensity score matching

PSM=propensity score matching. AA=Associate in Arts. BMI=body-mass index. PIR=family income-to-poverty ratio. eGFR=estimated glomerular filtration rate. GED=General Educational Development.

Variable		Before PSM			After PSM		
		Without eGFR decline (N=1281)	eGFR decline (N=1873)	*P* value	Without eGFR decline (N=913)	eGFR decline (N=913)	*P* value
Age (years)							
	<60	1023 (79.9%)	736 (39.3%)	<0.001	657 (72.0%)	647 (70.9%)	0.641
	≥60	258 (20.1%)	1137 (60.7%)		256 (28.0%)	266 (29.1%)	
PIR							
	<1.3	510 (39.8%)	525 (28.0%)	<0.001	326 (35.7%)	330 (36.1%)	0.86
	1.3–3.5	527 (41.1%)	839 (44.8%)		376 (41.2%)	365 (40.0%)	
	≥3.5	244 (19.0%)	509 (27.2%)		211 (23.1%)	218 (23.9%)	
BMI (kg/m^2)							
	<25	354 (27.6%)	416 (22.2%)	<0.001	221 (24.2%)	224 (24.5%)	0.941
	25–30	399 (31.1%)	682 (36.4%)		306 (33.5%)	299 (32.7%)	
	≥30	528 (41.2%)	775 (41.4%)		386 (42.3%)	390 (42.7%)	
Education							
	Less than high school	358 (27.9%)	471 (25.1%)	<0.001	235 (25.7%)	241 (26.4%)	0.386
	High-Grad/GED	363 (28.3%)	482 (25.7%)		244 (26.7%)	219 (24.0%)	
	Some College/AA	403 (31.5%)	588 (31.4%)		307 (33.6%)	305 (33.4%)	
	College/above	157 (12.3%)	332 (17.7%)		127 (13.9%)	148 (16.2%)	
Race							
	Non-Hispanic White	452 (35.3%)	1095 (58.5%)	<0.001	423 (46.3%)	402 (44.0%)	0.811
	Non-Hispanic Black	330 (25.8%)	323 (17.2%)		194 (21.2%)	201 (22.0%)	
	Mexican American	239 (18.7%)	179 (9.6%)		123 (13.5%)	131 (14.3%)	
	Other Hispanic	145 (11.3%)	140 (7.5%)		93 (10.2%)	89 (9.7%)	
	Other Race	115 (9.0%)	136 (7.3%)		80 (8.8%)	90 (9.9%)	
Sex							
	Female	423 (33.0%)	647 (34.5%)	0.396	287 (31.4%)	295 (32.3%)	0.725
	Male	858 (67.0%)	1226 (65.5%)		626 (68.6%)	618 (67.7%)	
Diabetes							
	Yes	177 (13.8%)	370 (19.8%)	<0.001	133 (14.6%)	153 (16.8%)	0.221
	No	1104 (86.2%)	1503 (80.2%)		780 (85.4%)	760 (83.2%)	
Smoke							
	Yes	687 (53.6%)	1047 (55.9%)	0.222	515 (56.4%)	518 (56.7%)	0.925
	No	594 (46.4%)	826 (44.1%)		398 (43.6%)	395 (43.3%)	
Hypertension							
	Yes	415 (32.4%)	1023 (54.6%)	<0.001	356 (39.0%)	369 (40.4%)	0.566
	No	866 (67.6%)	850 (45.4%)		557 (61.0%)	544 (59.6%)	
Alcohol							
	Yes	1011 (78.9%)	1314 (70.2%)	<0.001	718 (78.6%)	717 (78.5%)	1
	No	270 (21.1%)	559 (29.8%)		195 (21.4%)	196 (21.5%)	
Gun exposure							
	Yes	653 (51.0%)	1024 (54.7%)	0.0448	509 (55.8%)	504 (55.2%)	0.851
	No	628 (49.0%)	849 (45.3%)		404 (44.2%)	409 (44.8%)	
Work noise							
	Yes	799 (62.4%)	1026 (54.8%)	<0.001	569 (62.3%)	551 (60.4%)	0.414
	No	482 (37.6%)	847 (45.2%)		344 (37.7%)	362 (39.6%)	
Life noise							
	Yes	291 (22.7%)	382 (20.4%)	0.129	216 (23.7%)	220 (24.1%)	0.869
	No	990 (77.3%)	1491 (79.6%)		697 (76.3%)	693 (75.9%)	

Table S7. Hearing test of NHANES database

Note: *Code "666" was seen as 120 dB.

Variable	Data description	Variable number	Range
Right ear(0.5kHz)	https://wwwn.cdc.gov/Nchs/Nhanes/2009-2010/AUX_F.htm	AUXU500R	Value (-10~115) dB
	https://wwwn.cdc.gov/Nchs/Nhanes/2011-2012/AUX_G.htm	AUXU500R	Value (-10~90) dB
	https://wwwn.cdc.gov/Nchs/Nhanes/2015-2016/AUX_I.htm	AUXU500R	Value (-10~110) dB
	https://wwwn.cdc.gov/Nchs/Nhanes/2017-2018/AUX_J.htm	AUXU500R	Value (-10~105) dB
Left ear(0.5kHz)	https://wwwn.cdc.gov/Nchs/Nhanes/2009-2010/AUX_F.htm	AUXU500L	Value (-10~110) dB
	https://wwwn.cdc.gov/Nchs/Nhanes/2011-2012/AUX_G.htm	AUXU500L	Value (-10~100) dB
	https://wwwn.cdc.gov/Nchs/Nhanes/2015-2016/AUX_I.htm	AUXU500L	Value (-10~100) dB
	https://wwwn.cdc.gov/Nchs/Nhanes/2017-2018/AUX_J.htm	AUXU500L	Value (-10~105) dB
Right ear(1kHz)	https://wwwn.cdc.gov/Nchs/Nhanes/2009-2010/AUX_F.htm	AUXU1K1R	Value (-10~120) dB
	https://wwwn.cdc.gov/Nchs/Nhanes/2011-2012/AUX_G.htm	AUXU1K1R	Value (-10~100) dB
	https://wwwn.cdc.gov/Nchs/Nhanes/2015-2016/AUX_I.htm	AUXU1K1R	Value (-10~120) dB
	https://wwwn.cdc.gov/Nchs/Nhanes/2017-2018/AUX_J.htm	AUXU1K1R	Value (-10~105) dB
Left ear(1kHz)	https://wwwn.cdc.gov/Nchs/Nhanes/2009-2010/AUX_F.htm	AUXU1K1L	Value (-10~110) dB
	https://wwwn.cdc.gov/Nchs/Nhanes/2011-2012/AUX_G.htm	AUXU1K1L	Value (-10~100) dB
	https://wwwn.cdc.gov/Nchs/Nhanes/2015-2016/AUX_I.htm	AUXU1K1L	Value (-10~110) dB
	https://wwwn.cdc.gov/Nchs/Nhanes/2017-2018/AUX_J.htm	AUXU1K1L	Value (-10~100) dB
Right ear (2kHz)	https://wwwn.cdc.gov/Nchs/Nhanes/2009-2010/AUX_F.htm	AUXU2KR	Value (-10~115) dB
	https://wwwn.cdc.gov/Nchs/Nhanes/2011-2012/AUX_G.htm	AUXU2KR	Value (-10~120) dB
	https://wwwn.cdc.gov/Nchs/Nhanes/2015-2016/AUX_I.htm	AUXU2KR	Value (-10~105) dB
	https://wwwn.cdc.gov/Nchs/Nhanes/2017-2018/AUX_J.htm	AUXU2KR	Value (-10~110) dB
Left ear (2kHz)	https://wwwn.cdc.gov/Nchs/Nhanes/2009-2010/AUX_F.htm	AUXU2KL	Value (-10~120) dB
	https://wwwn.cdc.gov/Nchs/Nhanes/2011-2012/AUX_G.htm	AUXU2KL	Value (-10~120) dB
	https://wwwn.cdc.gov/Nchs/Nhanes/2015-2016/AUX_I.htm	AUXU2KL	Value (-10~115) dB
	https://wwwn.cdc.gov/Nchs/Nhanes/2017-2018/AUX_J.htm	AUXU2KL	Value (-10~105) dB
Right ear (3kHz)	https://wwwn.cdc.gov/Nchs/Nhanes/2009-2010/AUX_F.htm	AUXU3KR	Value (-10~120) dB
	https://wwwn.cdc.gov/Nchs/Nhanes/2011-2012/AUX_G.htm	AUXU3KR	Value (-10~120) dB
	https://wwwn.cdc.gov/Nchs/Nhanes/2015-2016/AUX_I.htm	AUXU3KR	Value (-10~105) dB
	https://wwwn.cdc.gov/Nchs/Nhanes/2017-2018/AUX_J.htm	AUXU3KR	Value (-10~105) dB
Left ear (3kHz)	https://wwwn.cdc.gov/Nchs/Nhanes/2009-2010/AUX_F.htm	AUXU3KL	Value (-10~120) dB

	https://wwwn.cdc.gov/Nchs/Nhanes/2011-2012/AUX_G.htm	AUXU3KL	Value (-10~120) dB
	https://wwwn.cdc.gov/Nchs/Nhanes/2015-2016/AUX_I.htm	AUXU3KL	Value (-10~120) dB
	https://wwwn.cdc.gov/Nchs/Nhanes/2017-2018/AUX_J.htm	AUXU3KL	Value (-10~110) dB
Right ear (4kHz)	https://wwwn.cdc.gov/Nchs/Nhanes/2009-2010/AUX_F.htm	AUXU4KR	Value (-10~120) dB
	https://wwwn.cdc.gov/Nchs/Nhanes/2011-2012/AUX_G.htm	AUXU4KR	Value (-10~120) dB
	https://wwwn.cdc.gov/Nchs/Nhanes/2015-2016/AUX_I.htm	AUXU4KR	Value (-10~120) dB
	https://wwwn.cdc.gov/Nchs/Nhanes/2017-2018/AUX_J.htm	AUXU4KR	Value (-10~110) dB
Left ear (4kHz)	https://wwwn.cdc.gov/Nchs/Nhanes/2009-2010/AUX_F.htm	AUXU4KL	Value (-10~120) dB
	https://wwwn.cdc.gov/Nchs/Nhanes/2011-2012/AUX_G.htm	AUXU4KL	Value (-10~120) dB
	https://wwwn.cdc.gov/Nchs/Nhanes/2015-2016/AUX_I.htm	AUXU4KL	Value (-10~120) dB
	https://wwwn.cdc.gov/Nchs/Nhanes/2017-2018/AUX_J.htm	AUXU4KL	Value (-10~110) dB
Right ear (6kHz)	https://wwwn.cdc.gov/Nchs/Nhanes/2009-2010/AUX_F.htm	AUXU6KR	Value (-10~120) dB
	https://wwwn.cdc.gov/Nchs/Nhanes/2011-2012/AUX_G.htm	AUXU6KR	Value (-10~120) dB
	https://wwwn.cdc.gov/Nchs/Nhanes/2015-2016/AUX_I.htm	AUXU6KR	Value (-10~120) dB
	https://wwwn.cdc.gov/Nchs/Nhanes/2017-2018/AUX_J.htm	AUXU6KR	Value (-10~100) dB
Left ear (6kHz)	https://wwwn.cdc.gov/Nchs/Nhanes/2009-2010/AUX_F.htm	AUXU6KL	Value (-10~120) dB
	https://wwwn.cdc.gov/Nchs/Nhanes/2011-2012/AUX_G.htm	AUXU6KL	Value (-10~120) dB
	https://wwwn.cdc.gov/Nchs/Nhanes/2015-2016/AUX_I.htm	AUXU6KL	Value (-10~120) dB
	https://wwwn.cdc.gov/Nchs/Nhanes/2017-2018/AUX_J.htm	AUXU6KL	Value (-10~105) dB

Table S8 Hearing related questionnaires of NHANES database

Variable	Description	Variable number	Definition of the code	
Self-perceived hearing loss	https://wwwn.cdc.gov/Nchs/Nhanes/2009-2010/AUQ_F.htm	AUQ131	Yes (Code=3~6)	No (Code=1~2)
	https://wwwn.cdc.gov/Nchs/Nhanes/2011-2012/AUQ_G.htm	AUQ054	Yes (Code=3~6)	No (Code=1~2)
	https://wwwn.cdc.gov/Nchs/Nhanes/2015-2016/AUQ_I.htm	AUQ054	Yes (Code=3~6)	No (Code=1~2)
	https://wwwn.cdc.gov/Nchs/Nhanes/2017-2018/AUQ_J.htm	AUQ054	Yes (Code=3~6)	No (Code=1~2)
Life noise exposure	https://wwwn.cdc.gov/Nchs/Nhanes/2009-2010/AUQ_F.htm	AUQ231	Yes (Code=1)	No (Code=2)
	https://wwwn.cdc.gov/Nchs/Nhanes/2011-2012/AUQ_G.htm	AUQ370	Yes (Code=1)	No (Code=2)
	https://wwwn.cdc.gov/Nchs/Nhanes/2015-2016/AUQ_I.htm	AUQ370	Yes (Code=1)	No (Code=2)
	https://wwwn.cdc.gov/Nchs/Nhanes/2017-2018/AUQ_J.htm	AUQ370	Yes (Code=1)	No (Code=2)
Gun noise exposure	https://wwwn.cdc.gov/Nchs/Nhanes/2009-2010/AUQ_F.htm	AUQ211	Yes (Code=1)	No (Code=2)
	https://wwwn.cdc.gov/Nchs/Nhanes/2011-2012/AUQ_G.htm	AUQ300	Yes (Code=1)	No (Code=2)
	https://wwwn.cdc.gov/Nchs/Nhanes/2015-2016/AUQ_I.htm	AUQ300	Yes (Code=1)	No (Code=2)
	https://wwwn.cdc.gov/Nchs/Nhanes/2017-2018/AUQ_J.htm	AUQ300	Yes (Code=1)	No (Code=2)
Work noise exposure	https://wwwn.cdc.gov/Nchs/Nhanes/2009-2010/AUQ_F.htm	AUQ290	Yes (Code=1)	No (Code=2)
	https://wwwn.cdc.gov/Nchs/Nhanes/2011-2012/AUQ_G.htm	AUQ350	Yes (Code=1)	No (Code=2)
	https://wwwn.cdc.gov/Nchs/Nhanes/2015-2016/AUQ_I.htm	AUQ350	Yes (Code=1)	No (Code=2)
	https://wwwn.cdc.gov/Nchs/Nhanes/2017-2018/AUQ_J.htm	AUQ350	Yes (Code=1)	No (Code=2)

Table S9. Other variables of NHANES database

Note: *For the convenience of calculation, Code "80" was regarded as 80 years old

Variable	Description	Variable number	Definition of the code	
Age	https://wwwn.cdc.gov/Nchs/Nhanes/2009-2010/DEMO_F.htm	RIDAGEYR	Value (0~79) (years)	≥ 80 years (80)
	https://wwwn.cdc.gov/Nchs/Nhanes/2011-2012/DEMO_G.htm	RIDAGEYR	Value (0~79) (years)	≥ 80 years (80)
	https://wwwn.cdc.gov/Nchs/Nhanes/2015-2016/DEMO_I.htm	RIDAGEYR	Value (0~79) (years)	≥ 80 years (80)
	https://wwwn.cdc.gov/Nchs/Nhanes/2017-2018/DEMO_J.htm	RIDAGEYR	Value (0~79) (years)	≥ 80 years (80)
Sex	https://wwwn.cdc.gov/Nchs/Nhanes/2009-2010/DEMO_F.htm	RIAGENDR	Male (Code=1)	Female (Code=2)
	https://wwwn.cdc.gov/Nchs/Nhanes/2011-2012/DEMO_G.htm	RIAGENDR	Male (Code=1)	Female (Code=2)
	https://wwwn.cdc.gov/Nchs/Nhanes/2015-2016/DEMO_I.htm	RIAGENDR	Male (Code=1)	Female (Code=2)
	https://wwwn.cdc.gov/Nchs/Nhanes/2017-2018/DEMO_J.htm	RIAGENDR	Male (Code=1)	Female (Code=2)
Race	https://wwwn.cdc.gov/Nchs/Nhanes/2009-2010/DEMO_F.htm	RIDRETH1	Mexican American (Code=1); Other Hispanic (Code=2); Non-Hispanic White (Code=3); Non-Hispanic Black (Code=4); Other Race - Including Multi-Racial (Code=5)	
	https://wwwn.cdc.gov/Nchs/Nhanes/2011-2012/DEMO_G.htm	RIDRETH1		
	https://wwwn.cdc.gov/Nchs/Nhanes/2015-2016/DEMO_I.htm	RIDRETH1		
	https://wwwn.cdc.gov/Nchs/Nhanes/2017-2018/DEMO_J.htm	RIDRETH1		
Education level	https://wwwn.cdc.gov/Nchs/Nhanes/2009-2010/DEMO_F.htm	DMDEDUC2	Less than 9th grade (Code=1);9-11th grade (Code=2); High school graduate/GED or equivalent (Code=3); Some college or AA degree (Code=4)	
	https://wwwn.cdc.gov/Nchs/Nhanes/2011-2012/DEMO_G.htm	DMDEDUC2		
	https://wwwn.cdc.gov/Nchs/Nhanes/2015-2016/DEMO_I.htm	DMDEDUC2		
	https://wwwn.cdc.gov/Nchs/Nhanes/2017-2018/DEMO_J.htm	DMDEDUC2		
PIR	https://wwwn.cdc.gov/Nchs/Nhanes/2009-2010/DEMO_F.htm	INDFMPIR	Values (0~4.99)	≥ 5.00 (5)
	https://wwwn.cdc.gov/Nchs/Nhanes/2011-2012/DEMO_G.htm	INDFMPIR	Values (0~4.99)	≥ 5.00 (5)
	https://wwwn.cdc.gov/Nchs/Nhanes/2015-2016/DEMO_I.htm	INDFMPIR	Values (0~4.99)	≥ 5.00 (5)
	https://wwwn.cdc.gov/Nchs/Nhanes/2017-2018/DEMO_J.htm	INDFMPIR	Values (0~4.99)	≥ 5.00 (5)
Alcohol use	https://wwwn.cdc.gov/Nchs/Nhanes/2009-2010/ALQ_F.htm	ALQ101	Yes (Code=1)	No (Code=2)
	https://wwwn.cdc.gov/Nchs/Nhanes/2011-2012/ALQ_G.htm	ALQ101	Yes (Code=1)	No (Code=2)
	https://wwwn.cdc.gov/Nchs/Nhanes/2015-2016/ALQ_I.htm	ALQ101	Yes (Code=1)	No (Code=2)
	https://wwwn.cdc.gov/Nchs/Nhanes/2017-2018/ALQ_J.htm	ALQ121	Yes (Code=1~7)	No (Code=0,8~10)
Diabetes	https://wwwn.cdc.gov/Nchs/Nhanes/2009-2010/DIQ_F.htm	DIQ010	Yes (Code=1)	No (Code=2)
	https://wwwn.cdc.gov/Nchs/Nhanes/2011-2012/DIQ_G.htm	DIQ010	Yes (Code=1)	No (Code=2)
	https://wwwn.cdc.gov/Nchs/Nhanes/2015-2016/DIQ_I.htm	DIQ010	Yes (Code=1)	No (Code=2)
	https://wwwn.cdc.gov/Nchs/Nhanes/2017-2018/DIQ_J.htm	DIQ010	Yes (Code=1)	No (Code=2)
Smoking	https://wwwn.cdc.gov/Nchs/Nhanes/2009-2010/SMQ_F.htm	SMQ020	Yes (Code=1)	No (Code=2)
	https://wwwn.cdc.gov/Nchs/Nhanes/2011-2012/SMQ_G.htm	SMQ020	Yes (Code=1)	No (Code=2)

	https://wwwn.cdc.gov/Nchs/Nhanes/2015-2016/SMQ_I.htm	SMQ020	Yes (Code=1)	No (Code=2)
	https://wwwn.cdc.gov/Nchs/Nhanes/2017-2018/SMQ_J.htm	SMQ020	Yes (Code=1)	No (Code=2)
Hypertension	https://wwwn.cdc.gov/Nchs/Nhanes/2009-2010/BPQ_F.htm	BPQ020	Yes (Code=1)	No (Code=2)
	https://wwwn.cdc.gov/Nchs/Nhanes/2011-2012/BPQ_G.htm	BPQ020	Yes (Code=1)	No (Code=2)
	https://wwwn.cdc.gov/Nchs/Nhanes/2015-2016/BPQ_I.htm	BPQ020	Yes (Code=1)	No (Code=2)
	https://wwwn.cdc.gov/Nchs/Nhanes/2017-2018/BPQ_J.htm	BPQ020	Yes (Code=1)	No (Code=2)
BMI	https://wwwn.cdc.gov/Nchs/Nhanes/2009-2010/BMX_F.htm	BMXBMI	Value (12.58~84.87) kg/m^2	
	https://wwwn.cdc.gov/Nchs/Nhanes/2011-2012/BMX_G.htm	BMXBMI	Value (12.4~82.1) kg/m^2	
	https://wwwn.cdc.gov/Nchs/Nhanes/2015-2016/BMX_I.htm	BMXBMI	Value (11.5~67.3) kg/m^2	
	https://wwwn.cdc.gov/Nchs/Nhanes/2017-2018/BMX_J.htm	BMXBMI	Value (12.3~86.2) kg/m^2	
Serum creatinine	https://wwwn.cdc.gov/Nchs/Nhanes/2009-2010/BIOPRO_F.htm	LBXSCR	Value (0.14~15.66) mg/dL	
	https://wwwn.cdc.gov/Nchs/Nhanes/2011-2012/BIOPRO_G.htm	LBXSCR	Value (0.16~9.51) mg/dL	
	https://wwwn.cdc.gov/Nchs/Nhanes/2015-2016/BIOPRO_I.htm	LBXSCR	Value (0.3~10.71) mg/dL	
	https://wwwn.cdc.gov/Nchs/Nhanes/2017-2018/BIOPRO_J.htm	LBXSCR	Value (0.25~12.74) mg/dL	

Table S10. Hearing questionnaire and hearing test of Tongren cohort

1. Hearing questionnaire			
Question: Have you perceived that you have hearing impairment?			
Answer:	Yes		
	No		

2. Hearing test	Frequency	Hearing loss	Normal
Hearing test was conducted by using pure tone air conduction audiometry			
	0.5kHz	>25db	≤25db
Whether participants had heard at 25db in specific frequencies	1kHz	>25db	≤25db
	2kHz	>25db	≤25db
	4kHz	>25db	≤25db

Table S11. Univariate Cox regression and multivariate Cox regression of the association between variables and eGFR decline in Tongren cohort.
HR=hazard ratio. CI=confidence interval. BMI=body-mass index. eGFR=estimated glomerular filtration rate.

Variable		Hearing impairment	univariable HR (CI95%, *P* value)	multivariable HR (CI95%, *P* value)
		No (N=4688)		
Age (years)	Mean ± SD	38.3 ± 8.9	1.08 (1.07-1.09, p<0.001)	1.09 (1.08-1.10, p<0.001)
Sex	male	2690 (57.4%)		
	female	1998 (42.6%)	0.45 (0.35-0.58, p<0.001)	0.50 (0.38-0.65, p<0.001)
BMI (kg/m²)	Mean ± SD	24.4 ± 4.1	1.05 (1.03-1.08, p<0.001)	1.03 (1.00-1.06, p=0.095)
Hypertension	No	4231 (90.3%)		
	Yes	457 (9.7%)	1.53 (1.14-2.05, p=0.0042)	0.60 (0.44-0.83, p=0.0020)
Noise exposure	No	4606 (98.3%)		
	Yes	82 (1.7%)	0.46 (0.15-1.44, p=0.184)	1.29 (0.99-1.69, p=0.061)
eGFR (ml/min/1.73m²)	>90	3300 (70.4%)		
	60-90	1327 (28.3%)	1.47 (1.16-1.86, p=0.0012)	0.85 (0.67-1.09, p=0.201)
	<60	61 (1.3%)	7.70 (3.39-17.49, p<0.001)	4.07 (1.79-9.24, p<0.001)

Table S12. Univariate Cox regression and multivariate Cox regression of the association between variables and eGFR decline in Tongren cohort.

HR=hazard ratio. CI=confidence interval. BMI=body-mass index. eGFR=estimated glomerular filtration rate.

Variable		eGFR decline	univariable HR (CI95%, *P* value)	multivariable HR (CI95%, *P* value)
		No (N=4064)		
Age (years)	Mean ± SD	38.7 ± 9.6	1.01 (1.01-1.02, p<0.001)	1.01 (1.01-1.02, p<0.001)
Sex	male	2381 (58.6%)		
	female	1683 (41.4%)	0.78 (0.69-0.87, p<0.001)	0.82 (0.73-0.92, p=0.0012)
BMI (kg/m²)	Mean ± SD	24.4 ± 4.2	1.02 (1.01-1.04, p<0.001)	1.01 (1.00-1.02, p=0.180)
Hypertension	No	3598 (88.5%)		
	Yes	466 (11.5%)	1.05 (0.90-1.23, p=0.540)	
Noise exposure	No	3967 (97.6%)		
	Yes	97 (2.4%)	1.10 (0.80-1.53, p=0.548)	
Self-perceived hearing loss	No	3794 (93.4%)		
	Yes	270 (6.6%)	1.49 (1.22-1.82, p<0.001)	1.31 (1.07-1.61, p=0.009)

Table S13. Univariate Cox regression and multivariate Cox regression of the association between variables and CKD in Tongren cohort.
HR=hazard ratio. CI=confidence interval. BMI=body-mass index. eGFR=estimated glomerular filtration rate. CKD=chronic kidney disease.

Variable		CKD	univariable HR (CI95%, *P* value)	multivariable HR (CI95%, *P* value)
		No (N=4064)		
Age (years)	Mean ± SD	38.7 ± 9.6	1.00 (1.00-1.01, p=0.424)	
Sex	male	2381 (58.6%)		
	female	1683 (41.4%)	0.73 (0.63-0.84, p<0.0001)	0.81 (0.69-0.95, p=0.009)
BMI (kg/m²)	Mean ± SD	24.4 ± 4.2	1.04 (1.02-1.06, p<0.0001)	1.03 (1.01-1.05, p=0.0006)
Hypertension	No	3598 (88.5%)		
	Yes	466 (11.5%)	1.08 (0.89-1.32, p=0.439)	
Noise exposure	No	3967 (97.6%)		
	Yes	97 (2.4%)	1.16 (0.76-1.76, p=0.487)	
Self-perceived hearing loss	No	3794 (93.4%)		
	Yes	270 (6.6%)	1.55 (1.20-2.00, p=0.0006)	1.49 (1.15-1.91, p=0.002)

TableS14. Association between hearing improvement and eGFR change among Chinese adults in Tongren cohort.

Values of eGFR change	Crude model		Model 1		Model 2	
	OR (95%CI)	*P* value	OR (95%CI)	*P* value	OR (95%CI)	*P* value
**All participants**	1.01(1.00- 1.02)	0.23	1.01(1.00- 1.02)	0.16	1.01(1.00- 1.02)	0.16
**Male**	1.00(0.99-1.01)	0.63	1.00(0.99- 1.01)	0.50	1.00(0.99-1.02)	0.48
**Female**	1.08(1.01-1.17)	**0.047**	1.08(1.02- 1.18)	**0.042**	1.10(1.02- 1.21)	**0.037**

Crude model: no cofounder.

Model 1: adjusted for body mass index (at diagnosis) and hypertension.

Model 2: adjusted for body mass index (at diagnosis), hypertension, follow-up time and BMI change.
y mass, index, the family of poverty ratio, diabetes, hypertension, and physical activity
OR: odds ratio; CI: confidence interval.

Figure S1. Association between eGFR and hearing threshold in different frequency hearing tests based on NHANES database

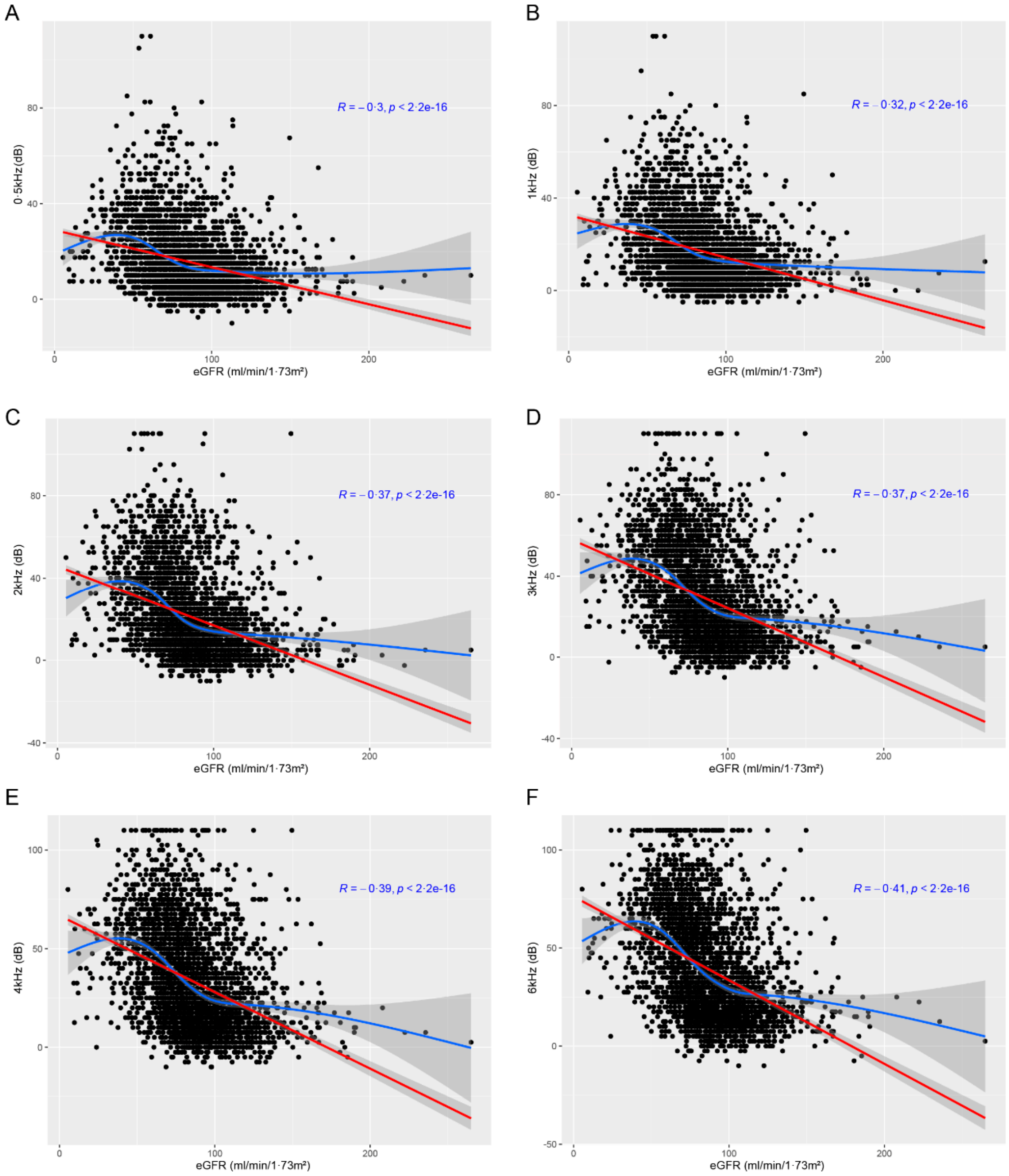

The correlation between average hearing threshold of bilateral ears at (A) 0.5 kHz (r=–0.3, p<0.0001), (B) 1 kHz (r=–0.32, p<0.0001), (C) 2 kHz (r=–0.37, p<0.0001), (D) 3 kHz (r=–0.37, p<0.0001), (E) 4 kHz (r=–0.39, p<0.0001) and (F) 6 kHz (r=–0.41, p<0.0001). eGFR, estimated glomerular filtration rate.

Figure S2. Absolute standardized mean difference of the process of propensity score matching in the NHANES population

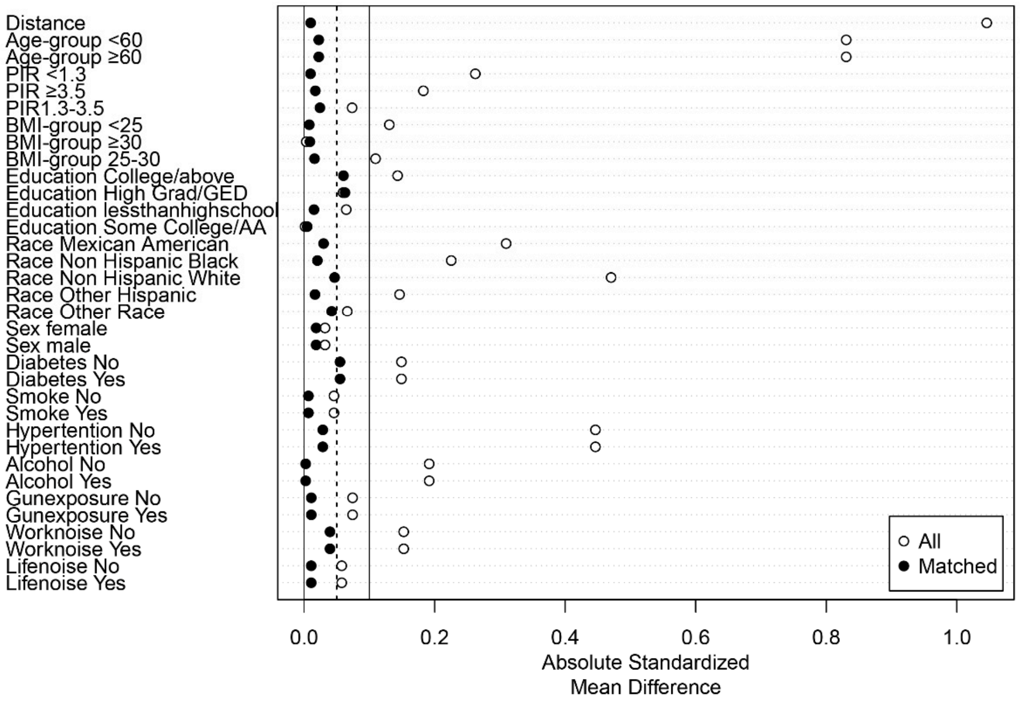

Figure S3. Distribution of propensity scores of unmatched and matched units

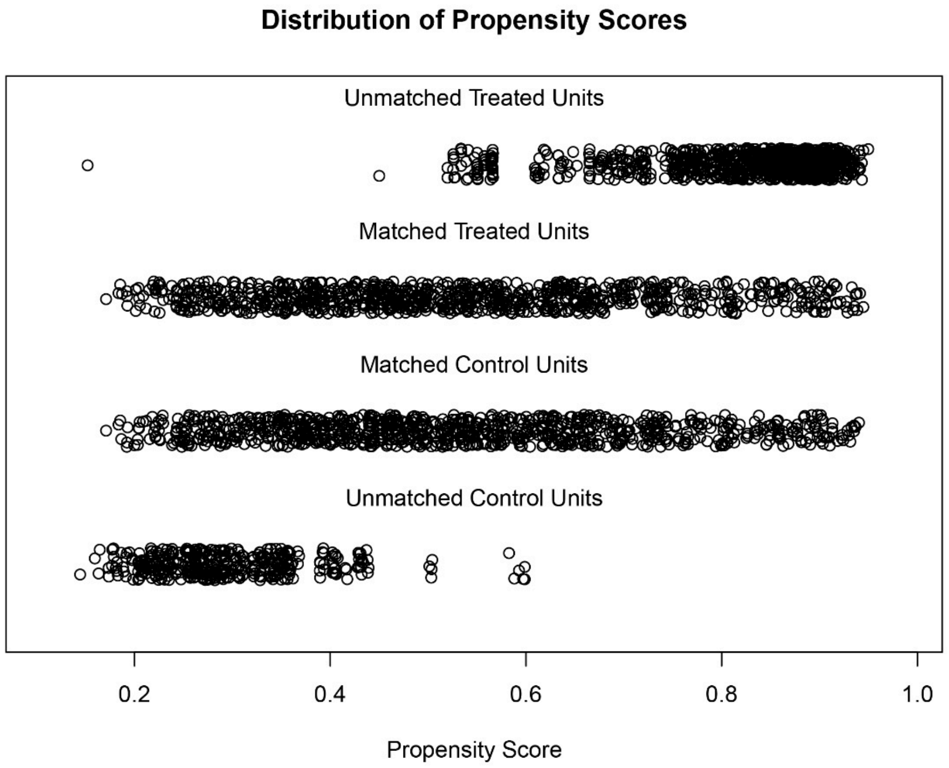

Figure S4. Proportion of the propensity score of unmatched and matched group

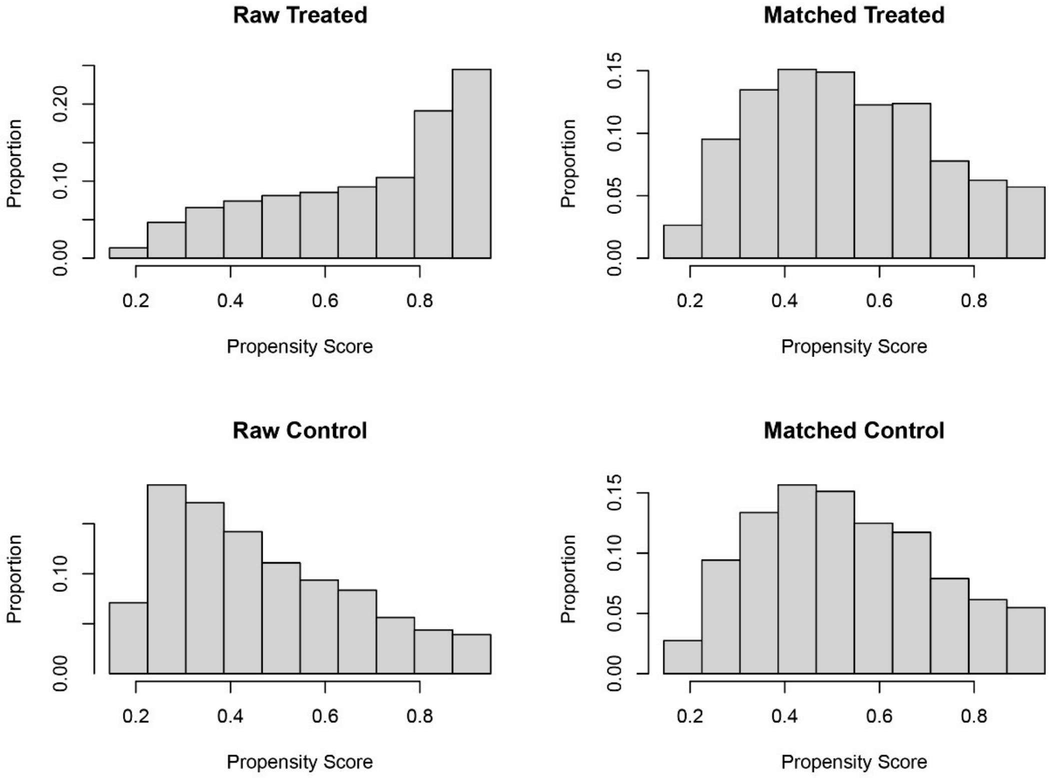

Figure S5. Comparison of binaural average hearing loss threshold at different frequency hearing test thresholds after propensity score matching based on whether eGFR was decreased

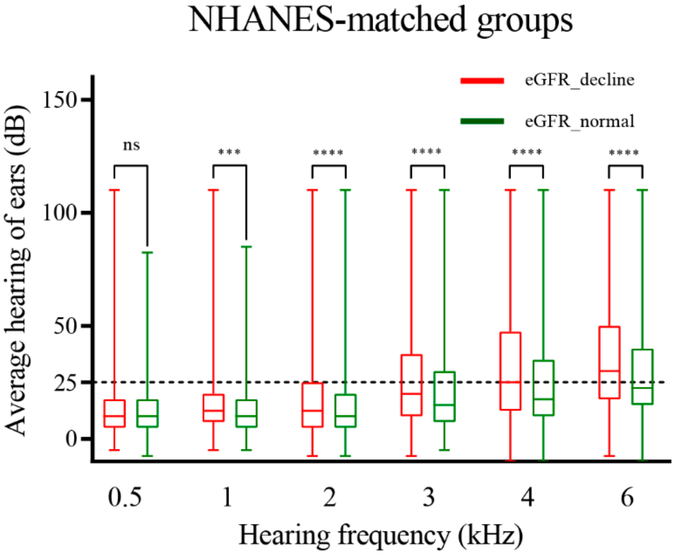

eGFR=estimated glomerular filtration rate

Figure S6. Comparison of different eGFR statuses in NHANES participants with and without self-perceived hearing loss

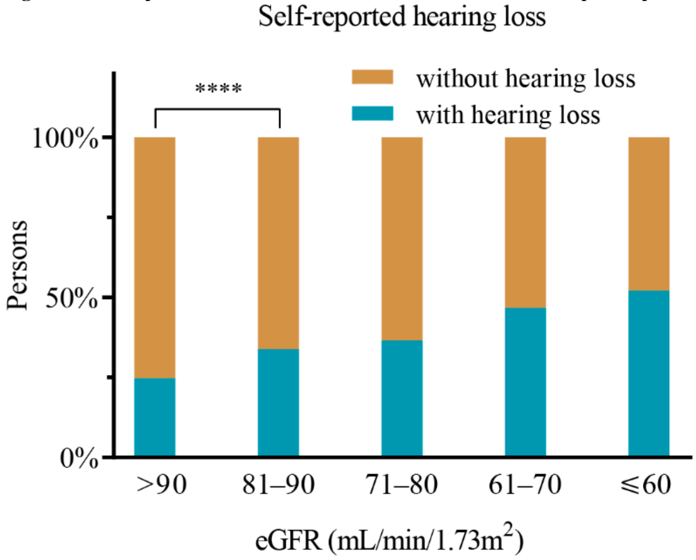

eGFR=estimated glomerular filtration rate.
